# Evolution from XIST-Independent to XIST-Controlled X-Chromosome Inactivation: Epigenetic Modifications in Distantly Related Mammals

**DOI:** 10.1371/journal.pone.0019040

**Published:** 2011-04-25

**Authors:** Julie Chaumeil, Paul D. Waters, Edda Koina, Clément Gilbert, Terence J. Robinson, Jennifer A. Marshall Graves

**Affiliations:** 1 Comparative Genomics Group, Evolution Ecology and Genetics, Research School of Biology, The Australian National University, Canberra, Australian Capital Territory, Australia; 2 Evolutionary Genomics Group, Department of Zoology, University of Stellenbosch, Matieland, South Africa; CNRS, France

## Abstract

X chromosome inactivation (XCI) is the transcriptional silencing of one X in female mammals, balancing expression of X genes between females (XX) and males (XY). In placental mammals non-coding *XIST* RNA triggers silencing of one X (Xi) and recruits a characteristic suite of epigenetic modifications, including the histone mark H3K27me3. In marsupials, where *XIST* is missing, H3K27me3 association seems to have different degrees of stability, depending on cell-types and species. However, the complete suite of histone marks associated with the Xi and their stability throughout cell cycle remain a mystery, as does the evolution of an ancient mammal XCI system. Our extensive immunofluorescence analysis (using antibodies against specific histone modifications) in nuclei of mammals distantly related to human and mouse, revealed a general absence from the mammalian Xi territory of transcription machinery and histone modifications associated with active chromatin. Specific repressive modifications associated with XCI in human and mouse were also observed in elephant (a distantly related placental mammal), as was accumulation of *XIST* RNA. However, in two marsupial species the Xi either lacked these modifications (H4K20me1), or they were restricted to specific windows of the cell cycle (H3K27me3, H3K9me2). Surprisingly, the marsupial Xi was stably enriched for modifications associated with constitutive heterochromatin in all eukaryotes (H4K20me3, H3K9me3). We propose that marsupial XCI is comparable to a system that evolved in the common therian (marsupial and placental) ancestor. Silent chromatin of the early inactive X was exapted from neighbouring constitutive heterochromatin and, in early placental evolution, was augmented by the rise of *XIST* and the stable recruitment of specific histone modifications now classically associated with XCI.

## Introduction

In therian (marsupial and placental) mammals, dosage compensation of X-linked genes in XX females and XY males is achieved by transcriptional silencing of X chromosome in females [1], [2] (reviewed by [3]). This process, called X chromosome inactivation (XCI), is perhaps the most striking example of epigenetic transcriptional regulation. Inactivation is established during early embryonic development [4], [5], when the inactive X chromosome (Xi) acquires many chromatin changes that transform it into transcriptionally silent, facultative heterochromatin. Notably, this process involves histone modifications and variants, DNA methylation, non-coding RNAs and differential nuclear compartmentalization (For reviews see [3], [6], [7]). How such a complex regulatory system evolved in mammals remains a mystery.

Nearly all studies of the molecular mechanism of X inactivation have been conducted on mice and humans, species representing a single clade of placental mammals (Euarchontoglires [8]). In these two species, initiation and propagation of inactivation is controlled *in cis* by a complex locus called the X inactivation centre. This locus contains the *XIST* gene (present in all placental mammal genomes; [9]) that produces the non-coding RNA responsible for triggering silencing [10], [11]. The accumulation of *XIST* RNA along the X chromosome chosen for inactivation is the first observable event in the XCI process, and is closely followed by several changes in chromatin (reviewed in [12]). The human and mouse inactive X rapidly loses histone modifications associated with transcription (H3K4me2, H3K9ac, H4Kac; **Table 1**), and gains specific repressive modifications (H3K9me2, H3K27me3, H4K20me1, H2AK119ub; **Table 1**) [4], [13], [14], [15], [16], [17], [18], [19], [20], [21], [22], [23], [24]. The role of these repressive marks is not fully understood, but they may be involved in the stabilization and somatic heritability of the silent state [20]. Little is known about the recruitment of these modifications, except that *Xist* RNA seems to recruit the Polycomb complex PRC2 responsible for H3K27me3 [17], [18], [20], and the protein(s) responsible for H4K20me1 [20].

**Table 1:** Profiles of repressive histone modifications associated with constitutive or facultative heterochromatin in placental and marsupial mammals.

			Placental mammal Xi		
	Modification	Constitutive heterochromatin	XIST dependent	XIST independent	Marsupial Xi
Repressive marks	H3K9me2	−	+	−	30%
	H3K27me3	−	+	−	30%
	H4K20me1	−	+	−	−
	H2AK119ub	−	+	−	?
	H3K9me3	+	−	+	+
	H3K27me1	+	−	−	−
	H4K20me3	+	−	+	+
Active marks	H3K4me	−	−	−	−
	H3K9ac	−	−	−	−
	H4Kac	−	−	−	−

Novel insight into the mechanism of placental mammal XCI can be gained by comparing human and mouse epigenetic components to those of distantly related mammals. Work on the distantly related afrotherian mammals (placental mammals such as the elephant) revealed an Xi displaying classic features of XCI (i.e. Barr body formation and late replication compared to the active X [25], [26]) and bearing the *XIST* gene [9], suggesting that many features of placental mammal XCI were established before the placental (eutherian) radiation.

Marsupial (metatherian) mammals are even more distantly related to humans and mice, having diverged from placental mammals 148MYA. XCI also occurs in marsupials. There are several molecular and phenotypic differences between marsupial XCI, and human and mouse XCI, which offers important insights into how this process works, and how it evolved. Like the Xi in human and mouse, the marsupial Xi replicates late in S phase [27], [28], and sex chromatin (Barr body) has been observed in some species and tissues [29]. However, contrary to human and mouse, marsupial XCI has been described as incomplete, tissue-specific [30], and unstable [31]. Differential histone H4 acetylation was observed between the two X chromosomes in fibroblasts from female marsupials [32], but no locus specific DNA methylation differences were detected [33], [34]. However, global DNA hypomethylation was recently detected on Xi in possum (*Trichosurus vulpecula*) and potoroo (*Potorous tridactylus*) at metaphase [35].

One striking difference is that marsupial XCI is imprinted, with the paternal X chromosome (X^p^) always chosen for inactivation [28], [36], whereas X inactivation is random in placental mammals. Interestingly, the paternally derived X is also chosen for inactivation during the first stages of mouse development, and remains inactive in extra-embryonic tissues [4], [37], [38].

This led to the suggestion that marsupial imprinted XCI could represent an ancestral inactivation mechanism.

Perhaps the most fundamental difference between marsupial and placental XCI is the absence of *XIST* from the marsupial genome [9], [39], [40], raising many intriguing questions about the evolution of this remarkable system, and challenging the importance of *XIST* in initiation and/or maintenance of the inactive state.

In this study, we wished to discover whether the histone marks associated with XCI in human and mouse were also found in distantly related placental mammals, and, in the absence of the *XIST* gene, in marsupials. Previously we showed [41] that there was a depletion of active chromatin marks on the mitotic inactive X chromosome in female fibroblast cells of the tammar wallaby, but with no apparent accumulation of inactive histone modifications. However, metaphase is not an ideal model for the study of epigenetic features underpinning XCI, as it represents only a fraction of the cell cycle with a specific condensed state of chromatin, and may not represent histone modifications on Xi in the rest of the cell cycle. A recent report showed a H3K27me3 enrichment on opossum Xi at different frequencies in brain and liver tissues of opossum (*Monodelphis domestica*) [42]. Here we conducted a detailed, extensive and comparative analysis of the pattern of active and repressive histone marks in interphase nuclei of phylogenetically important mammalian species: two marsupial models (tammar wallaby and opossum) and an afrotherian (elephant). We observed a specific pattern of inactive marks on the marsupial Xi, with a variable degree of stability throughout cell cycle, and strikingly different from the pattern seen in all placental mammals studied so far, including the elephant (examined herein). These results shed new light on the evolution of X chromosome inactivation and the role of the *XIST* gene.

## Results

### Profile of H3K27me3 on the inactive X chromosome of distantly related mammals

In placental mammals (human/mouse) the inactive X chromosome (Xi) can be easily recognized by detecting *XIST* RNA accumulation with RNA FISH, or by immuno-staining of the tri-methylation of lysine 27 of histone H3 (H3K27me3) [17], [18], [20].

#### Elephant

**Figure 1:**
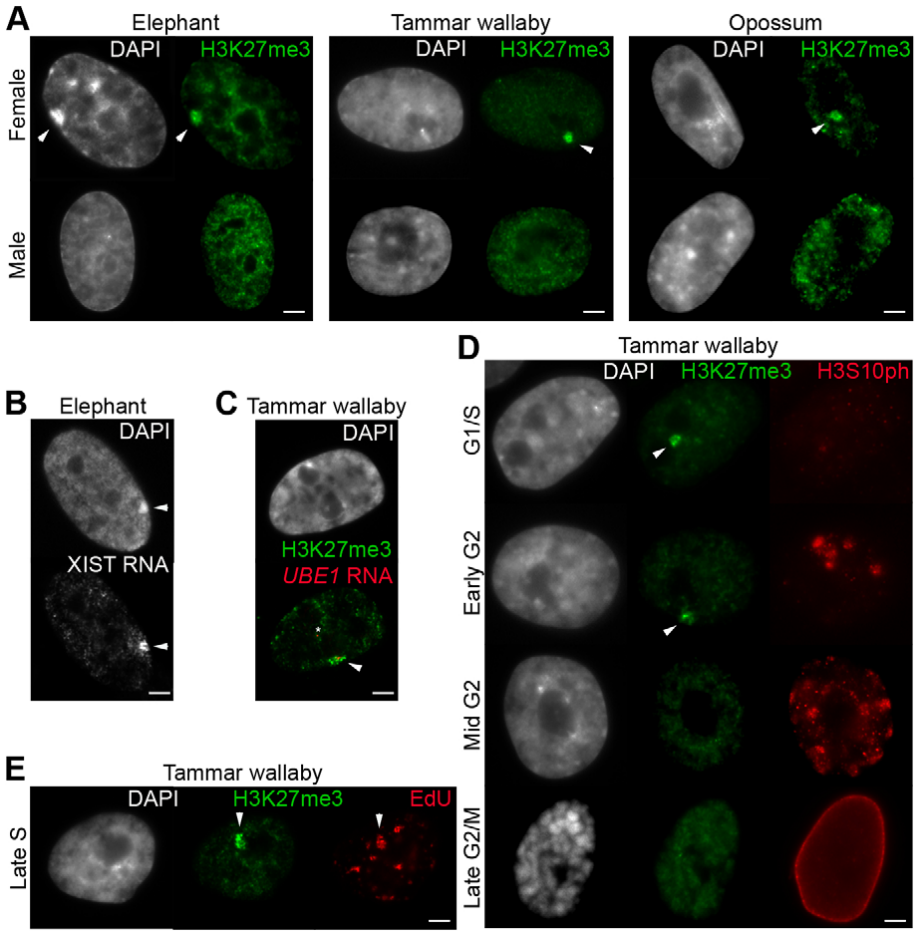
Status of H3K27me3 enrichment and XIST-RNA accumulation on the inactive-X in distantly related mammals. **A**. Examples of immunofluorescence for H3K27me3 (in green) on elephant cells (left panel), tammar wallaby cells (centre) and opossum cells (right panel). The top row shows a representative nucleus from female cell, the bottom row from male cell. Arrow heads point the inactive X chromosome enriched in H3K27me3. n = 100. **B**. Example of RNA FISH showing accumulation of *XIST* transcripts (in grey) on the DAPI-dense inactive X chromosome of female elephant cells. **C**. Immunofluorescence for H3K27me3 (in green) combined with RNA FISH for the *UBE1* primary transcripts (in red) in female cells from tammar wallaby. Arrow head points the inactive X chromosome containing *UBE1* transcripts and enriched in H3K27me3. Asterisk shows *UBE1* transcripts from the active X chromosome. n = 30. **D**. Dual immunofluorescence for H3K27me3 (in green) and H3S10ph (in red) on female tammar wallaby cells showing that enrichment in H3K27me3 (arrow heads) on the Xi is restricted to the G1/S phase and the early G2 phase of the cell cycle. n = 90. **E**. Immunofluorescence for H3K27me3 (in green) combined with a cell proliferation assay to label replicating DNA (in red) showing H3K27me3 enrichment on the Xi in late S phase, at the time of Xi replication (enrichment of H3K27me3 (green) on the replicating Xi (red)). n = 200. DAPI is shown in grey. Scale bar = 1 µm.

We performed immunofluorescence (IF) against H3K27me3 on interphase nuclei of male and female elephant cells. A nuclear territory was enriched in H3K27me3 in 98% of female but not male nuclei (n = 100 for both sexes) (**Figure 1A**, left panel). In all cells this domain corresponded to a DAPI dense region of the nucleus, identical to the previously identified Barr body in female elephant cells [25]. Moreover, using RNA FISH, we showed that this Barr body was decorated by *XIST* transcripts (**Figure 1B**). Thus the inactive X chromosome of female basal placental mammals, like other placentals, harbours a stable accumulation of *XIST* RNA and H3K27me3, demonstrating that this system was established before the divergence of Afrotheria from other placental mammals 105 MYA [43].

#### Marsupials

In order to find a cytological marker for the marsupial Xi, we performed IF for H3K27me3 on interphase nuclei of male and female primary fibroblasts of two marsupial representatives, the tammar wallaby and the opossum. Primary cultures were studied at low passage (<5), with two independent replicates for the tammar wallaby. We used three independent and extensively tested antibodies to confirm our results (one polyclonal [Upstate-Millipore] and two monoclonal [Abcam and [19]]; **Table 2**). In both marsupial species a large nuclear domain was strikingly enriched for H3K27me3 in 30% of nuclei from female, but not male cells (n = 100 for both sexes, for each primary culture and for each antibody) (**Figure 1A**, centre and right panels). This domain of H3K27me3 enrichment was often (∼65%) but not always associated with a DAPI-dense domain, suggesting that this enrichment is not only the result of chromatin compaction (see examples in **Figures 1** **,** **2** **and** **3**).

**Figure 2:**
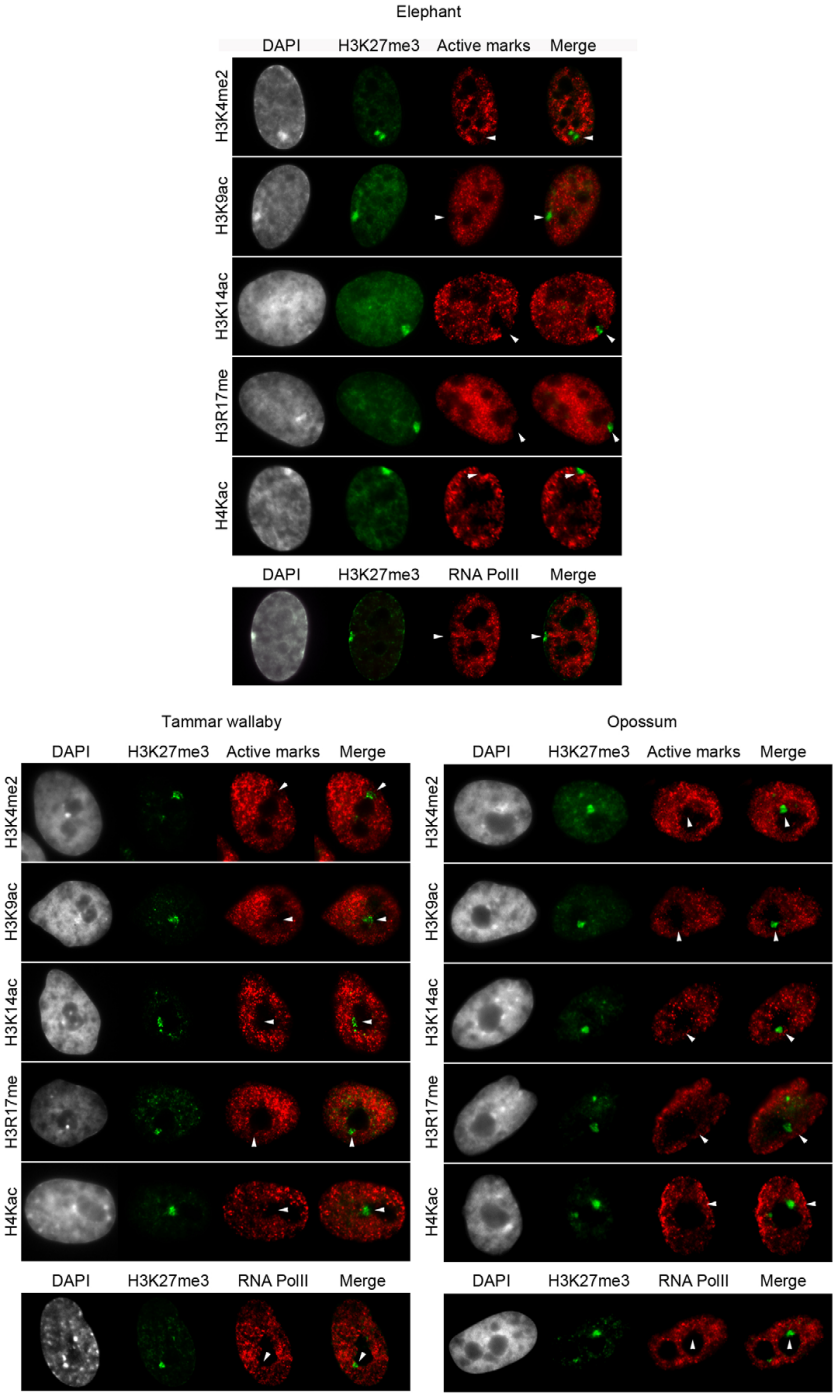
Depletion of active marks and RNA polymerase II on the inactive-X of therian mammals. Dual immunofluorescence with H3K27me3 (in green) combined with H3K4me2, H3K9ac, H3K27me1, H3K14ac, H3R17me2, H4Kac or with RNA polymerase II (in red) in elephant (top), tammar wallaby (bottom left) and opossum (bottom right) female cells. n = 100–200. DAPI is shown in grey. Scale bar = 1 µm.

**Figure 3:**
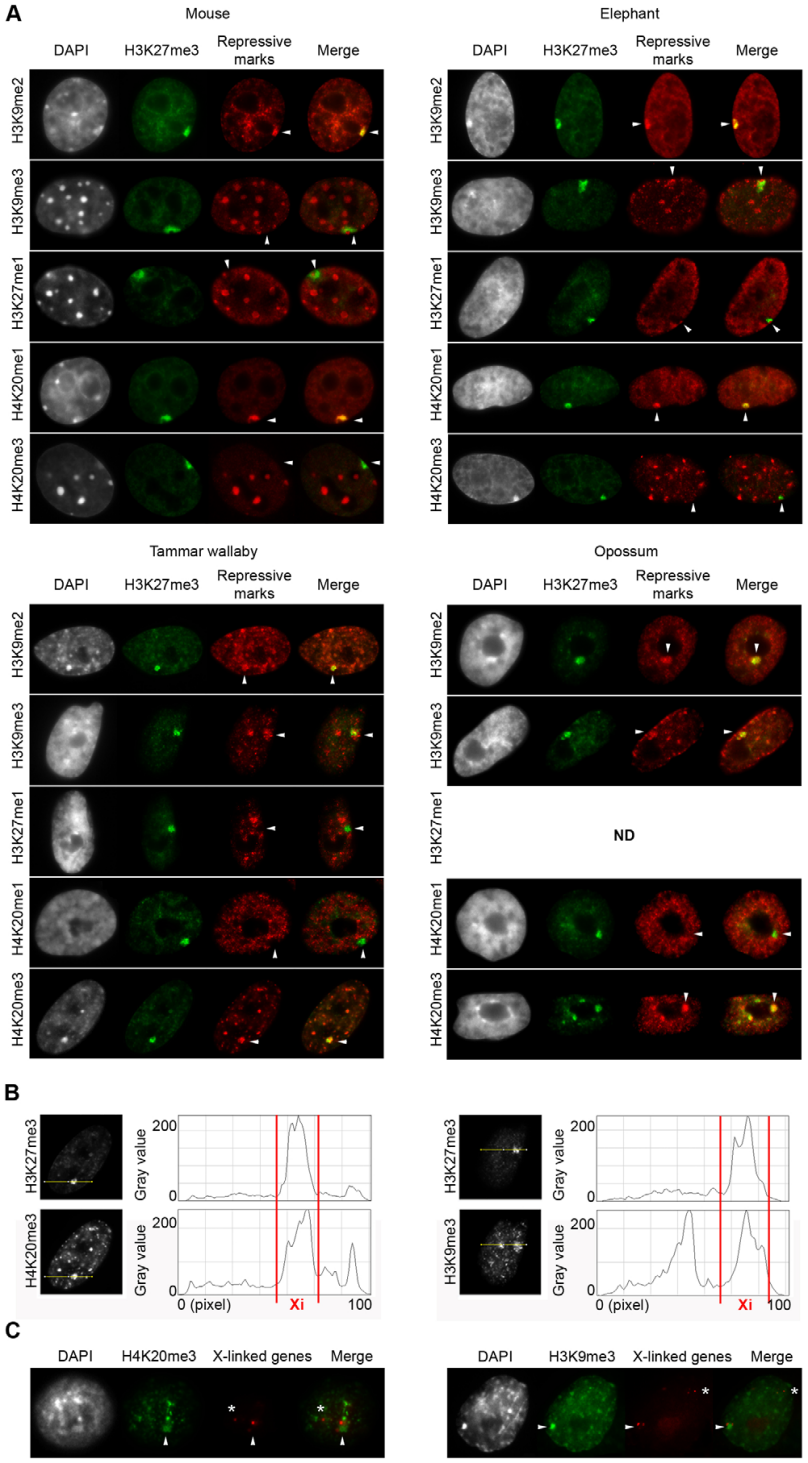
Patterns of repressive marks on the inactive-X of distantly related mammals. **A**. Dual immunofluorescence with H3K27me3 (in green) combined with H3K9me2, H3K9me3, H3K27me1, H4K20me1 or H4K20me3 (in red) in mouse (top left), elephant (top right), tammar wallaby (bottom left) and opossum (bottom right) female cells. n = 100. **B**. Line scans of H3K27m3, H4K20me3 (left) and H3K9me3 (right) intensities in the Xi territory. **C**. Sequential immunofluorescence for H4K20me3 (in green; left) or H3K9me3 (in green; right) followed with a DNA FISH for 3 X-linked genes *UBE1*, *ATRX* and *MECP2* (in red) in tammar wallaby female cells. The arrow head indicates the inactive X chromosome enriched in H3K27me3, asterisks indicates the genes from the active X chromosome. n = 50. DAPI is shown in grey. Scale bar = 1 µm.

**Table 2:** Primary antibodies used for the immunofluorescence experiments.

Antibody	Dilution	Species	Supplier
RNA Polymerase II (CTD4H8)	1/200	Mouse	Upstate-Millipore (05-623)
Histone H3K4me2	1/200	Rabbit	Upstate-Millipore (07-030)
Histone H3K9ac	1/100	Rabbit	Upstate-Millipore (07-352)
Histone H3K9me2	1/200	Rabbit	Upstate-Millipore (07-441)
Histone H3K9me3	1/300	Rabbit	Upstate-Millipore (07-442)
Histone H3K14ac	1/200	Rabbit	Upstate-Millipore (07-353)
Histone H3R17me	1/200	Rabbit	Upstate-Millipore (07-214)
Histone H3K27me1	1/300	Rabbit	Upstate-Millipore (07-448)
Histone H3K27me3	1/200	Rabbit	Upstate-Millipore (07-449)
Histone H3K27me3	1/200	Mouse	Abcam (6147)
Histone H3K27me2/3	1/200	Mouse	Rougeulle et al., 2004
Histone H4Kac	1/200	Rabbit	Upstate-Millipore (06-946)
Histone H4K5ac	1/100	Rabbit	Upstate-Millipore (07-327)
Histone H4K8ac	1/200	Rabbit	Upstate-Millipore (07-328)
Histone H4K12ac	1/300	Rabbit	Upstate-Millipore (07-761)
Histone H4K20me1	1/300	Rabbit	Upstate-Millipore (07-440)
Histone H4K20me3	1/200	Rabbit	Upstate-Millipore (07-463)
Histone H3S10ph	1/500	Rabbit	Upstate-Millipore (06-570)

To confirm that this domain was indeed the Xi in the tammar wallaby, we performed IF combined with RNA FISH using a BAC containing *UBE1*, an X-linked gene that largely escapes X inactivation in wallaby (i.e. is bi-allelically expressed in ∼70% of nuclei; [44]). When *UBE1* was bi-allelically expressed in cells showing a large H3K27me3 enrichment domain, one of the two signals was located at the border of this domain, confirming that it represented one X chromosome (n = 30) (**Figure 1C**). When it was mono-allelically expressed the single signal was never associated with the H3K27me3 domain, confirming that the X enriched in H3K27me3 was the Xi.

#### Cell cycle specificity of H3K27me3 enrichment on the marsupial Xi

This H3K27me3 enrichment on the Xi in 30% of marsupial cells (contrasting to almost 100% in placental mammals), as well as the failure to detect it on wallaby mitotic chromosomes [41], led us to hypothesize that H3K27me3 accumulation is cell cycle specific. Because primary cells are sensitive to artificial treatment, we used techniques that identified cell cycle stage (rather than cell synchronization) to minimize the risk of interfering with normal accumulation (or loss) of transient modifications.

We first used phosphorylation of serine 10 of histone H3 (H3S10ph) as a marker of cell cycle phases (**Table 2**). This modification, involved in chromatin condensation, is absent in G1/S and starts to associate with few centromeres in early G2, all centromeres in mid G2, and then spreads to the whole arms of chromosomes in late G2/mitosis [45]. H3K27me3 enrichment was visible in 80% of early G2 phase nuclei, but never in mid to late G2 or mitosis (n = 90) (**Figure 1D**), supporting its absence on metaphase chromosomes [41]. H3K27me3 enrichment was also detected 13% of G1/S phase.

Therefore, we used a cell proliferation assay to label replicating DNA (Click-iT EdU Alexa Fluor 488, Invitrogen) to discriminate between G1 (no nuclear EdU staining), early S phase (overall nuclear EdU staining) and late S phase (EdU staining on centromeres/Xi). The G1/S phase enrichment of H3K27me3 was only detected in S phase, and never in G1 (n = 200). 20% of S phase enrichment was observed in early S phase, and 80% was observed in late S phase (**Figure 1E**). Thus, in marsupials, H3K27me3 begins to accumulate on Xi in early S phase, but is most prominent on Xi in late S and early G2 phase, around the time of Xi replication [27], [28]. Additionally, when H3K27me3 was enriched on Xi, it was located close to the nucleolus in more than 90% of the cases (see examples in **Figures 1** **,** **2** **and** **3**).

### Exclusion of histone modifications associated with transcription from the afrotherian and marsupial inactive X chromosome

In human and mouse the Xi is depleted for histone modifications associated with active chromatin (euchromatin) [4], [13], [14], [16], [46], [47], [48]. To determine whether this holds true for the afrotherian and marsupial Xi, we performed a series of dual immunofluorescence using H3K27me3 (as a marker for the Xi) in combination with four histone H3 modifications (H3K4me2, H3K9ac, H3K14ac and H3R17me), or histone H4 acetylation (global acetylation as well as single acetylation at K5, K8 and K12) (**Figure 2**, **Table 2**). We were also interested in whether or not Xi was lacking RNA Polymerase II, as was shown in mouse [49] (**Figure 2**).

We found that all these histone modifications and RNA Pol II were excluded from the Xi in elephant as well as marsupial female cells (100<n<200; **Figure 2**). In marsupial cells, these euchromatic markers were analysed relative to H3K27me3, i.e. in 30% of interphase cells. Unfortunately technical limitations prohibited the analysis of these active marks in 100% of interphase nuclei as H3K27me3 was the only suitable marker of the Xi available to us: Signals from the X-chromosome paint were faint and unreliable; immunofluorescence with the active marks followed by DNA FISH with X-linked genes resulted in punctate DNA FISH signals that were difficult to correlate with a hole in IF staining; finally, in the next section we describe two histone marks enriched on the marsupial Xi throughout most of the cell cycle, but unfortunately we could not perform combined IF as all the antibodies were polyclonal (raised in rabbit).

However, we confirm here the exclusion of these active marks from the Xi for at least 30% of interphase, which together with our previous study showing that they were also excluded from mitotic chromosomes [41], suggests that exclusion of active marks is a stable feature of the marsupial Xi throughout interphase and metaphase. Moreover, we were able to combine the IF of a stable repressive mark on Xi with RNA Polymerase II (as this antibody is monoclonal; see last section of result), which confirmed that RNA polymerase II was excluded from the Xi throughout the cell cycle. Thus, the stable formation of a transcriptionally inert nuclear compartment devoid of active marks and transcription machinery is a feature of XCI shared across all therian mammals.

### Profile of repressive histone marks on inactive X chromosome in distantly related mammals

In human and mouse, the Xi bears a characteristic signature of repressive histone modifications (XCI-marks: H3K9me2, H3K27me3, H4K20me1, H2AK119ub) (**Table 1**). This pattern of Xi-associated facultative heterochromatin differs strikingly from constitutive pericentromeric heterochromatin (PCH-marks: H3K9me3, H3K27me1 and H4K20me3) (**Table 1**) [50], [51], [52], [53]. However, a detailed analysis of human mitotic chromosomes revealed that the Xi is organised into non-overlapping bands, with some enriched in XCI-marks (associated with *XIST*-domains in interphase), and others enriched in PCH-marks (not associated with *XIST* and often restricted to centromere and telomeres) [54], [55]. The same alternate pattern was found in bovine species [56]. To investigate the status of the XCI and PCH-marks on the elephant and marsupial Xi, we conducted dual immunofluorescences with antibodies specific to six of these marks, in combination with H3K27me3 (**Table 2**, **Figure 3**) (NB: H2AK119ub and macroH2A were not analyzed because the antibodies available to us produced no consistent signal of enrichment, even in mouse cells).

#### Elephant

The histone modification pattern in female elephant cells was similar to that in mouse. The H3K27me3 domain was strongly enriched in H3K9me2 and H4K20me1 in more than 95% of cells (n = 100 for each antibody) (**Figure 3A**, top panels). H3K9me3, which is specific to PCH but is also associated with regions of the human and bovine Xi [54], [56], [57], similarly showed a slight enrichment on the Xi in a fraction of elephant cells. As in human and mouse, H3K27me1 and H4K20me3 were only associated with PCH.

#### Marsupials

The pattern of repressive histone marks on the marsupial Xi showed some similarities to other therians, but also displayed some striking differences (n = 100 for each antibody).

As in placental mammals, the Xi positive for H3K27me3 showed an enrichment of H3K9me2 and a depletion of H3K27me1. However, we unexpectedly observed that the marsupial Xi was depleted for H4K20me1; moreover, H4K20me3 and H3K9me3 (PCH-marks in placental mammals) were not only associated with PCH in our marsupial representatives, but were also strongly enriched on the Xi (**Figure 3A**, bottom panels; **Table 1**). Because in humans XCI and PCH-marks form non-overlapping regions on the Xi [54], we performed intensity line scans along the enrichment domains. These scans showed that the domains overlapped at least partially in the nucleus, suggesting that (in contrast to the human Xi) H3K27me3 and H3K9me3/H4K20me3 may be associated with the same sequences on the marsupial Xi (**Figure 3B**).

Because H3K27me3 is enriched on the marsupial Xi only in late S/early G2 phase, we analyzed whether H4K20me3 and H3K9me3 were also cell cycle dependent or more stably enriched. We performed immunofluorescence followed by DNA FISH (to locate the Xs) with three X-linked genes that are known to escape X inactivation in 40–70% of nuclei (*UBE1*, *ATRX* and *MECP2*) [44]. We consistently observed two clusters of DNA FISH signals (corresponding to the two Xs). In more than 90% of nuclei the H4K20me3 or H3K9me3 domain was invariably situated next to one of the two DNA FISH signals (n = 50) (Figure 3C). Therefore, because these cell lines were not synchronized, H3K9me3 and H4K20me3 must be enriched on Xi throughout at least 90% of the cell cycle. Thus, these two inactive marks, specific to pericentromeric heterochromatin in placental mammals, are stably associated with the Xi in marsupials. We confirmed that RNA Pol II was excluded from the Xi by performing dual IF (with H4K20me3 and RNA Pol II) before DNA FISH (data not shown). Finally, this experiment also demonstrated that *UBE1*, *ATRX* and *MECP2* were always located at the periphery of the repressive domain, as do genes escaping XCI in mice [49]. Thus, in marsupials, H3K27me3 and H3K9me2 are enriched on Xi in late S phase/early G2, whereas H4K20me3 and H3K9me3 are stably enriched on the marsupial Xi throughout the cell cycle.

## Discussion

In this study we examined the histone modification profiles of the inactive X chromosome in interphase cells of elephant (a representative of Afrotheria, a clade distantly related to human and mouse), and two marsupial representatives (wallaby and opossum) that represent the therian mammals most distantly related to placental mammals.

### Depletion of active chromatin marks and transcription machinery from the inactive X chromosome is common to all therian mammals

Here we demonstrated that the Xi of marsupials (and also the elephant) is stably depleted throughout cell cycle of histone marks associated with transcription, which is consistent with their absence from the marsupial Xi during mitosis [32], [35], [41]. We also showed that RNA polymerase II is excluded from the Xi. Thus, the inactive X chromosome in all therian mammals forms a transcriptionally inert nuclear compartment devoid of active histone marks and transcription machinery, and that this must be an ancestral epigenetic characteristic of the therian X inactivation system.

Loss of active histone marks, and exclusion of RNA polymerase II, has been ascribed directly or indirectly to the accumulation of *XIST* transcripts on the placental Xi. Formation of this repressive compartment must be an *XIST* independent characteristic of XCI in marsupial cells, a feature likely to have been present in the common ancestor to therian mammals. However, this does not necessarily mean that the loss of active histone marks from the Xi is *XIST*-independent in placental cells, even though it has been demonstrated that this loss of active marks was independent of the gene-silencing function of *Xist* RNA in mice ES cells [49]. One hypothesis is that there could be another non-coding RNA triggering the formation of the transcriptionally inert compartment in marsupial cells [42], the function of which was supplanted by the rise of *XIST* in a placental ancestor.

### XIST RNA accumulation and recruitment of inactive histone marks to the inactive X chromosome is conserved across all placental mammals

The elephant X chromosome was recently demonstrated to have the same gene content and order as the human X chromosome, with only a difference in centromere positioning [58]. This remarkably conserved gene order along the whole placental mammal X chromosome over such long evolutionary time (∼105 million years) contrasts with the marsupial X chromosome, which has suffered multiple rearrangements between opossum and wallaby over approximately 65 million years [59].

Genome sequencing revealed that the elephant genome contains *XIST* [9], dating the origin of this gene after the divergence of marsupials and placentals 145 MYA, but before the placental radiation 105MYA. Here we showed that the Xi is coated by *XIST* RNA and displays the same specific combination of repressive histone marks as in mouse and human (**Table 1**). This identical chromatin profile suggests that *XIST*-dependent XCI was fully established before the placental mammal radiation ∼105MY and has been maintained in all lineages (studied thus far) since then. Strong selection against rearrangements that disrupt this conserved *cis*-acting XCI machinery is consistent with the observed hyper-conservation of the placental mammal X chromosome [58].

### Association of the inactive X chromosome with the two XCI-specific repressive marks is cell cycle specific in tammar wallaby and opossum fibroblasts

As in placental mammals, the marsupial Xi is enriched for the histone modifications H3K27me3 and H3K9me2. However, although there was a strong enrichment of H3K27me3 it was only observed in 30% of female nuclei, in contrast to enrichment in almost 100% of nuclei in placental mammals. H3K9me2 was enriched less strongly than in placental cells, and only in conjunction with H3K27me3. We showed that enrichment was transient and occurred almost exclusively in late S phase and early G2, around the time when the inactive X is replicating [27], [28]. These results are consistent with our previous failure to detect these repressive marks on the inactive X during metaphase [41].

It was recently shown that H3K27me3 is enriched in 60% and 98% of interphase cells from opossum liver and brain respectively [42] which, together with our data showing enrichment in 30% of opossum and wallaby fibroblasts, suggests that the stability of H3K27me3 accumulation is tissue-specific and/or specie-specific in marsupials. A very recent study found H3K27me3 enriched on 50% of the mitotic Xi from Australian common brush-tail possum fibroblast cells [35]. This discrepancy with our current study (and previous study; [41]) may be explained by specie-specific accumulation of H3K27me3 on Xi (wallaby vs. brushtail possum). Nevertheless, all these studies demonstrate quite variable stability of H3K27me3 on the marsupial Xi in time (cell cycle stages) and space (cell types, species). This is not unexpected given that this mark is *XIST* dependent in placental mammals [17], [18], [20]. Even though it remains possible that H3K27me3 (and H3K9me2) is recruited by an unknown non-coding RNA in marsupials, these studies suggest that one important effect of the newly evolved *XIST* gene was to stabilize H3K27me3 recruitment to Xi.

It is interesting to note that most inactive X chromosomes enriched with H3K27me3 are associated with the nucleolus. It has been shown that in mouse cells, the Xi is targeted to the perinucleolar compartment in mid-to-late S phase, and that this could be involved in maintaining its heterochromatic state by facilitating recruitment of repressive marks, especially H3K27me3 [60]. Location of the marsupial Xi next to the nucleolus may also help define its epigenetic state; however, this mechanism is triggered by the *XIST* RNA in mice, so in the absence of *XIST* it is unclear how this mechanism operates in marsupials.

### Marsupial inactive X chromosome displays a profile of repressive marks similar to that of constitutive heterochromatin

Surprisingly, the marsupial inactive X is strongly and stably enriched in H3K9me3 and H4K20me3 (>90% of nuclei), marks that are generally associated with constitutive pericentromeric heterochromatin. This supports the recent findings that H3K9me3 and H4K20me3 is present on the mitotic brushtail possum Xi [35]. Thus, these marks are enriched on Xi throughout the entire cell cycle and, therefore, not transient. Moreover, our data suggests that the XCI-marks (H3K27me3) and PCH-marks (H3K9me3/H4K20me3) are organised differently to the mutually exclusive arrangement observed in human cells [54]; rather, they seem to occupy the same sub-nuclear compartment formed by the Xi. Conversely, the marsupial Xi showed no enrichment of H4K20me1, a modification that is enriched on the placental mammal Xi and which is consistent with studies suggesting that accumulation of this mark on Xi is *XIST* dependent [20]. Thus, the marsupial Xi displays a signature of repressive epigenetic marks very different to that of Xi-facultative heterochromatin associated with *XIST* RNA in placental mammals, and more similar to pericentromeric heterochromatin (**Table 1**).

### Evolution of transcriptionally silent chromatin on the inactive X chromosome

In this study we compare the epigenetic profiles of imprinted (marsupials) XCI to random (placental mammals) XCI in adult somatic cells. Female mouse cells also undergo a transient stage of imprinted paternal X inactivation during early development (the X^p^ is then reactivated in blastocysts for subsequent random inactivation but remains inactive in extra-embryonic tissues [4], [37], [38]). However, in contrast to the marsupial Xi, the inactive murine X^p^ does not harbour strong H4K20me3/H3K9me3 enrichment; instead its epigenetic signature is very similar to that observed in random placental mammal XCI. The constitutive heterochromatin marks associated with marsupial inactive X chromatin might, therefore, represent an ancestral epigenetic system of transcriptional silencing on Xi that was exapted from neighbouring constitutive heterochromatin.

**Figure 4:**
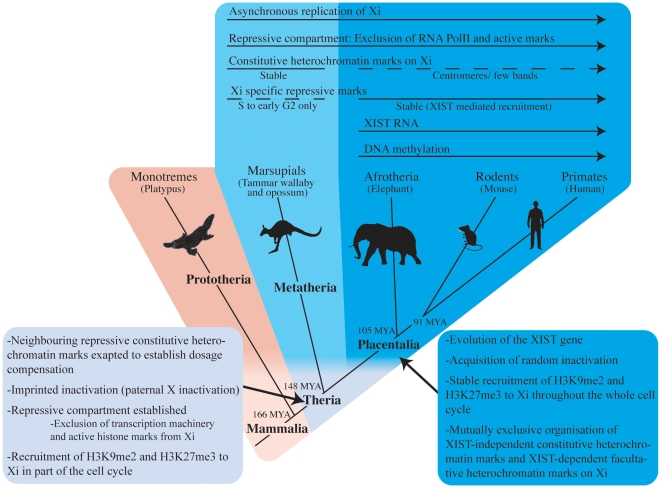
A model for the evolution of mammalian X-chromosome inactivation. Arrows above the phylogeny show epigenetic features underlying X chromosome inactivation (solid arrows indicate stable components of XCI within the clades, whereas dashed lines indicate unstable repressive modification). We propose that in the ancestral therian mammal neighbouring centromeric constitutive heterochromatin histone modifications were exapted to reduce transcription from the paternal X chromosome in females. H3K27me3 and H3K9me2 were also recruited to the Xi in a cell-cycle and/or cell-lineage dependent manner. After the divergence of placental from marsupial mammals, the *XIST* gene evolved and H3K27me3 and H3K9me2 (along with H4K20me1) were stably recruited into the XCI machinery. In placental mammals the original silencing machinery was restricted, and organised in mutually exclusive domains with the Xi-specific facultative heterochromatin. In marsupials, much of the original silencing machinery that was recruited from constitutive heterochromatin remains in place.

We propose a model for the evolution of the X-chromosome inactivation process whereby silencing of the paternal X chromosome in the therian mammal ancestor was achieved by the accumulation of constitutive heterochromatin marks, as well as H3K27me3 and H3K9me2 (**Figure 4**). The evolution of *XIST* in the placental mammal ancestor enabled these two marks (together with H4K20me1) to be stably recruited to establish new *XIST*-dependent X inactivation machinery. Thus, with strong epigenetic signatures of constitutive heterochromatin, and weaker signatures of Xi-specific facultative heterochromatin, the marsupial inactive X chromosome could represent an intermediate stage in the process of recruitment of constitutive heterochromatin epigenetic marks into early therian dosage compensation, to the more complex and stable *XIST*-dependent heterochromatin observed in placental mammals.

Our findings give new insight into the evolution of this complex epigenetic regulation of the X chromosome, and suggest new avenues of investigation to deepen our understanding of this crucial process. It will be particularly important to identify the enzymes responsible for the repressive marks on the marsupial Xi, how they are recruited to the Xi chromatin (another non-coding RNA?), and how the cell-cycle specific pattern of the recruitment of H3K27me3 operates (is it linked to replication and which enzyme(s) are involved?).

## Materials and Methods

### Ethics statement

The study was approved, and all samples were collected and held under The Australian National University Animal Experimentation Ethics Committee proposal numbers R.CG.11.06 and R.CG.14.08.

### Cell culture

Male and female primary fibroblast cell cultures from mouse (*Mus musculus*), tammar wallaby (*Macropus eugenii*), grey short-tailed opossum (*Monodelphis domestica*) and African savanna elephant (*Loxodonta africana*) were established from ear clips. Cells were cultured at 35°C or 37°C in 5% CO_2_ and 45% DME/45%Amniomax C100/10% fetal calf serum (Gibco, Invitrogen).

### Immunofluorescence and RNA FISH

Immunofluorescence on interphase cells was performed as previously described [61], [62]. Cells were grown on coverslips coated with 0.5% gelatin, then fixed in 3% paraformaldehyde/1× PSB for 10 minutes at room temperature (RT), permeabilized in 0.5% triton/1× PBS/(2 mM Vanadyl Ribonucleoside Complex was added when subsequent RNA FISH was to be performed) for 5 minutes on ice and blocked in 1% BSA/1× PBS (0.4 U/µl of RNAguard [Amersham/Pharmacia] was added when subsequent RNA FISH was to be performed). Cells were incubated with primary and secondary antibodies in blocking solution sequentially for 1 hour at room temperature in dark and humid chamber (see **Table 2**). After three washes in 1× PBS, coverslips were mounted onto slides with Vectashield® containing DAPI (Vector laboratories).

The replication assay was performed following the manufacturer's instructions (Click-iT EdU Alexa Fluor 488, Invitrogen). Cells were incubated for 10 minutes in the presence of 10 µM EdU just prior to detection.

For subsequent RNA FISH, preparations were post-fixed (after the last wash in 1× PBS) in 3% paraformaldehyde/1× PBS for 10 minutes at RT, then rinsed in 2× SSC before overnight hybridization with the probe at 37°C in a dark humidity chamber. A BAC containing *UBE1*, an X-linked gene, was used as a probe (Me_KBa-51D22; Arizona Genomics Institute, Tucson AZ, USA). It was labelled in a nick translation reaction with SpectrumOrange dUTP (Enzo Diagnostics, NY, USA) following manufacturer's instructions. 300 ng of labelled BAC probe was precipitated with 10 µg glycogen and 1 µg of *M. eugenii* sheared genomic DNA and resuspended in 15 µl of hybridization buffer (50% formamide, 20% dextran sulfate, 2× SSC, 1 mg/ml BSA, 10 mM Vanadyl Ribonucleoside Complex). The probe was then denatured for 10 min at 70°C and pre-annealed for 30 min at 37°C before overnight hybridization. Slides were washed three times in 50% formamide/2× SSC (adjusted to pH 7.2) and three times in 2× SSC for 5 min each at 37°C before being mounted with Vectashield® containing DAPI.

### Immunofluorescence combined with DNA FISH

We performed sequential immunofluorescence/DNA FISH experiments adapted from previous protocols [62]. Cells were grown on slides instead of coverslips. We performed immunofluorescences as described above and recorded images and coordinates of the cells using a Delta Vision microscope (see below). After image capture, the coverslips and mounting medium were removed by three washes of 0.2% Tween-20/4× SSC at 42°C. The slides were subsequently incubated with 10 U/mL RNase A in 2× SSC for 1 h at 37°C, dehydrated in an ethanol series, denatured in 70% formamide/2× SSC (pH 7.2) for 2–3 minutes at 75°C, dehydrated again, and hybridized with probes overnight at 42°C in a darkened humidity chamber. Slides were washed three times (5 min each) in 50% formamide/2× SSC (adjusted to pH 7.2), and three times (5 min each) in 2× SSC, at 42°C. Slides were mounted with Vectashield® containing DAPI.

BACs containing the X-linked genes *MECP2* (Me_VIA-143H14; Victorian Institute of Animal Science, Attwood, VIC, Australia), *ATRX* (Me_VIA-43E9) and *UBE1* were used as probes for DNA FISH. BAC DNA was labelled by nick translation with SpectrumOrange dUTP as described for RNA FISH. The three BAC probes were co-precipitated (300 ng each) and prepared for overnight hybridization as described above.

### XIST RNA FISH

Coverslips with male or female cells were fixed and permeabilized as described above. These were dehydrated in an ethanol series before hybridization with the *XIST* probe. The following primers were designed to amplify *XIST* exonic sequence from elephant genomic DNA.

LAF XIST 1f - AGTGTTAGTGACCCATTCCCTTTG

LAF XIST 1r - TCTTGGCATAGAGTTGTTGACCAG

LAF XIST 2f - GCTGTTCCTTATGCCACGCTAC

LAF XIST 2r - TCTGCCTTTTGTTCTCCTTCAGTC

LAF XIST 3f - GCCAAATAGGTGGACTGTGCC

LAF XIST 3r - TGCCTTGCTTTCCTCTCACG

LAF XIST 4f - GAACAGTAAAAGGGCTAAGGGTTTG

LAF XIST 4r – GCTCAAGTGTCTTCCTGACTCTAAGC

One primer pair (pair 1) amplified part of exon 4, and the remainder amplified different regions of exon 6. PCR conditions were: 94°C, 2′; followed by 30 cycles of 94°C, 30″/56°C, 30″/72°C, 30″. The four amplicons were sequenced to confirm identity. 50 ng of each product was labelled with SpectrumOrange dUTP in a PCR under the same cycling conditions described above. Labelled products were co-precipitated, the pellet resuspended in 10 µl of deionized formamide and then denatured at 75°C for 7 minutes. After cooling on ice for 2 minutes, 10 µl 2× hybridization buffer (4×SSC, 40% dextran sulphate, 2 mg/ml BSA, 10 mM Vanadyl Ribonucleoside Complex) was added. 10 µl of the probe was hybridised to a coverslip with female nuclei, and 10 µl to a coverslip with male nuclei, overnight at 37°C in a darkened and humidity chamber. After hybridization, coverslips were washed three times for 5 minutes each in 50% formamide/2×SSC at 42°C, and three times for 5 minutes each in 2×SSC at 42°C. Coverslips were rinsed with distilled water and mounted with Vectashield® containing DAPI.

### Microscopy and analyses

Two-dimensional microscopy was performed using a Zeiss Axiolan epifluorescence microscope. Images were captured on a SPOT RT Monochrome CCD (charge-coupled device) camera (Diagnostic Instruments Inc., Sterling Heights, MI, USA) and analyzed using IPLab imaging software (Scanalytics Inc., Fairfax, VA, USA).

Sequential three-dimension microscopy was performed using a Delta Vision microscope system (Applied precision, Issaquah, WA, USA). 3D stacks were acquired with z planes separated by 0.2 µm and deconvolved using the Softworx software algorithm (conservative ratio method, 7 iterations). For sequential immuno-DNA FISH experiments, nuclei were first imaged and their coordinates were recorded with the Softworx software after immunofluorescence to assist recaptured after DNA FISH.
